# Supramalleolar osteotomy combined with lateral ligament reconstruction and talofibular immobilization for varus ankle osteoarthritis with excessive talar tilt angle

**DOI:** 10.1186/s13018-019-1457-6

**Published:** 2019-11-28

**Authors:** Wenqing Qu, Dajiang Xin, Shengjie Dong, Wenliang Li, Yanping Zheng

**Affiliations:** 1 Qilu Hospital, Shandong University, 107# Wenhua Xi Road, Jinan, 250012 Shandong People’s Republic of China; 2 Department of Orthopaedics, Yantaishan Hospital, 91# Jiefang Road, Zhifu District, Yantai, 264001 Shandong People’s Republic of China; 3 Department of Orthopaedics, Qilu Hospital (Qingdao), Shandong University, 758# Hefei Road, Qingdao, 266035 Shandong People’s Republic of China

**Keywords:** Varus ankle osteoarthritis, Excessive talar tilt angle, Supramalleolar osteotomy, Lateral ligament reconstruction, Talofibular immobilization

## Abstract

**Background:**

Although supramalleolar osteotomy is the main joint-preserving method for the treatment of varus ankle osteoarthritis, it tends to be ineffective when ankle osteoarthritis presents in combination with an excessive talar tilt angle. The purpose of this study was to present a new surgical technique, supramalleolar osteotomy combined with lateral ligament reconstruction and talofibular immobilization, for the treatment of varus ankle osteoarthritis with an excessive talus tilt angle and to evaluate the clinical and radiological results.

**Methods:**

From January 2013 to October 2016, a total of 17 patients with 17 cases of varus ankle arthritis with excessive talar tilt angles (larger than 7.3°) underwent surgical treatment using our new technique. The American Orthopaedic Foot and Ankle Society (AOFAS) clinical ankle-hindfoot scale and a visual analogue scale (VAS) were used to evaluate ankle function and pain before surgery and at the last follow-up. The medial distal tibial angle (MDTA), anterior distal tibial angle (ADTA), talar tilt angle (TTA), and hindfoot moment arm values (HMAVs) were evaluated on weight-bearing radiographs acquired preoperatively and at the last follow-up.

**Results:**

The AOFAS score improved significantly from 45.8 ± 2.1 before surgery to 84.8 ± 1.8 after surgery (*p* < 0.001), and the VAS score decreased from 4.9 ± 0.4 to 1.1 ± 0.2 (*p* < 0.001). The MDTA, TTA, and HMAV changed from 80.9° ± 0.4° to 90.1° ± 0.4°, 11.7° ± 0.6° to 1.4° ± 0.3°, and 12.6 mm ± 0.8 mm to 4.2 mm ± 0.6 mm, respectively (each *p* < 0.001). The ADTA showed no obvious change (*p* = 0.370). The staging of 11 cases (65%) improved. Intramuscular vein thrombosis of the lower limbs occurred in 1 patient 1 week after surgery, and superficial infection occurred in 1 patient.

**Conclusions:**

Supramalleolar osteotomy combined with lateral ligament reconstruction and talofibular immobilization can correct the load of the weight-bearing ankle and effectively improve the ankle function. As the talar tilt angle can be significantly improved after surgery, this technique can be used for the treatment of varus ankle osteoarthritis with an excessive TTA.

## Background

Ankle osteoarthritis (OA) can be manifested as varus, valgus, or neutral alignment, in which varus accounts for approximately 52% of the cases [1, 2]. Abnormal alignment often leads to progressive aggravation of ankle osteoarthritis, which gradually progresses to end-stage ankle arthritis. At present, ankle fusion or arthroplasty is the main method for treating end-stage ankle arthritis. However, the former causes loss of ankle motion and the possibility of subsequent adjacent joint degeneration, and the latter may be difficult to perform in patients with preoperative ankle stiffness, high demand for postoperative exercise, osteoporosis, and so on [3, 4]. Therefore, it is of great significance to prevent the progression of moderate ankle arthritis to end-stage arthritis.

A deformation apex of varus ankle arthritis (the center of rotation of angulation, CORA) may peak at the distal tibia, tibia-talus joint, subtalar joint, or at the calcaneus. As the main joint-preserving surgery, supramalleolar osteotomy can adjust the alignment of the tibia and talus and normalize the stress distribution on the ankle joint surface, thus preventing degeneration of the joint; this technique is increasingly widely used [5–8]. Although traditional supramalleolar osteotomy has been reported to be effective [9–13], clinical and radiographic results showed that varus ankle arthritis with an excessive talar tilt angle (TTA) could not be treated effectively. Most authors considered this technique to be ineffective in correcting the TTA if the preoperative TTA was greater than 10° [10] or 7.3° [12]. Lee [14] designed a modified supramalleolar osteotomy in response to the problem. However, Ahn et al. [1] found that Lee’s osteotomy technique could improve most radiographic parameters satisfactorily, while there was no significant improvement in TTA.

Chronic lateral instability of the ankle (CLIA) can cause varus tilt of the talus and is considered to be the most common cause of varus ankle OA [2, 5]. In the treatment of varus ankle arthritis, lateral ligament reconstruction is performed with poor improvements to the TTA [8, 12]. We hypothesized that after lateral ligament reconstruction in supramalleolar osteotomy for the treatment of varus ankle arthritis, immobilization with Kirschner wires for 6 weeks to maintain accurate matching of the talus in the ankle mortise can correct the excessive TTA and achieve a favorable outcome. We investigated the results of this technique for the treatment of varus ankle arthritis with an excessive TTA.

## Materials and methods

### Patients

After Institutional Review Board approval (No. 2018CTEC-SL-008), we retrospectively reviewed our data from January 2013 to October 2016. The inclusion criteria were as follows: (1) stage 2 or stage 3 adult ankle OA according to Tanaka ankle arthritis staging [12], with clinical symptoms such as ankle swelling, pain, and limited movement; (2) a medial distal tibial angle (MDTA) less than 84° [15]; (3) a TTA larger than 7.3° [12]; and (4) a follow-up of more than 2 years. Patients with acute or chronic ankle infection, gout or rheumatoid arthritis, neoplastic arthropathy, Charcot arthropathy, neuromuscular dysfunction, lower limb deformity proximal to the ankle, severe ipsilateral subtalar arthritis, and clearly widened ankle mortise or varus deformity of the calcaneus body were excluded.

There were a total of 17 patients, including 13 men and 4 women with a mean age of 56.5 years (50 to 65). Fifteen patients had a history of severe ankle sprains, 5 had a history of diabetes, and 5 had a history of smoking. There were 2 patients in stage 2, 11 patients in stage 3a, and 4 patients in stage 3b.

### Clinical analysis

Clinical evaluations were conducted using the American Orthopaedic Foot and Ankle Society (AOFAS) clinical ankle-hindfoot scale [16] and a visual analogue scale (VAS) for pain. The AOFAS score included pain, function, and alignment of the affected limb, with a full score of 100. The VAS score ranged from 0 to 10, depending on the severity of the pain.

### Radiological analysis

**Fig. 1:**
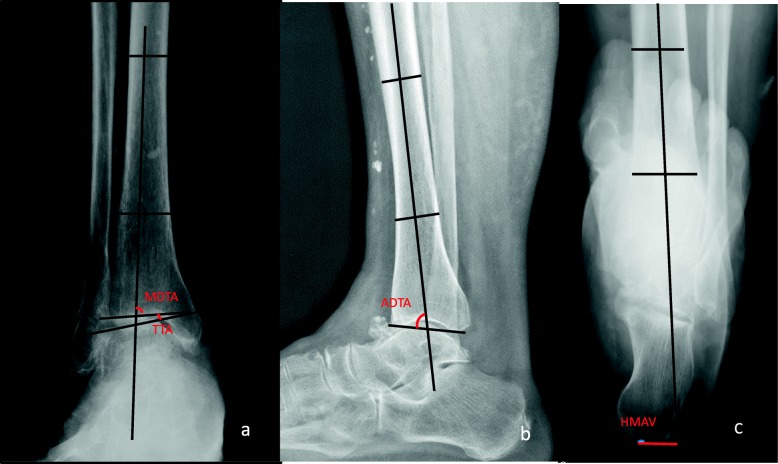
Female, 62 years old, right varus ankle arthritis, stage 3b. Preoperative weight-bearing anteroposterior radiograph showing a decreased MDTA and increased TTA from neutral (**a**). Preoperative weight-bearing lateral radiograph showing an increased ADTA from neutral (**b**). Preoperative weight-bearing hindfoot alignment radiograph showing an increased HMAV from neutral (**c**)

Preoperatively and at the last follow-up, weight-bearing radiographs in the anteroposterior view, lateral view, and hindfoot alignment view were applied for the following measurements (Fig. 1): (1) MDTA, the medial angle between the axis of the tibia and the tibial plafond on an anteroposterior radiograph [1]; (2) TTA, the angle between the tibial plafond and talar dome [1]; (3) the anterior distal tibial angle (ADTA), the anterior angle between the axis of the tibia and the tibial plafond on a lateral radiograph [1]; and (4) the hindfoot moment arm values (HMAVs), the distance between the most inferior point on the calcaneus and the tibial line on the hindfoot alignment view [17].

According to their histories of ankle instability, 6 patients underwent anteroposterior and lateral stress radiography. CT scans were performed for 11 patients to investigate the osteophytes and free bodies obstructing the motion of the joints.

### Surgical technique

All surgical procedures were performed by the first author (WQ) and his assistants. Under epidural or general anesthesia, the patient was placed in a supine position with a tourniquet placed on the proximal end of the leg. An anteromedial and an anterolateral incision were made to expose the ankle joint.

#### Joint debridement and distal tibial osteotomy

The osteophytes were debrided, and the anterior talofibular ligament (ATFL) and calcaneofibular ligament (CFL) were explored. Bone marrow stimulation was performed by cartilage drilling, if necessary [18]. The oblique distal tibial osteotomy procedure was the same as the technique introduced by Lee [14]. A line connecting a point on the medial tibial cortex 5 cm proximal to the ankle joint and a point on the lateral tibial cortex 5 mm proximal to the joint was marked by two parallel needles. After the distal tibia was cut using a sharp swing saw, a lamina spreader was inserted for gradual correction of the varus deformity, and the intact lateral tibial cortex was retained as the hinge. After satisfactorily correcting the MDTA and ADTA under fluoroscopy, two wedge-shaped autologous iliac bone grafts were inserted into the defect of the tibia, and an anatomical locking plate was used to complete the fixation. Consistent with Tanaka et al. [12], a fibula osteotomy was performed in 5 patients when intraoperative fluoroscopy showed that the space between the talus and the lateral malleolus was significantly narrowed while the osteotomy site of the tibia was gradually opened.

#### Reconstruction of the lateral ankle ligaments and talofibular immobilization

**Fig. 2:**
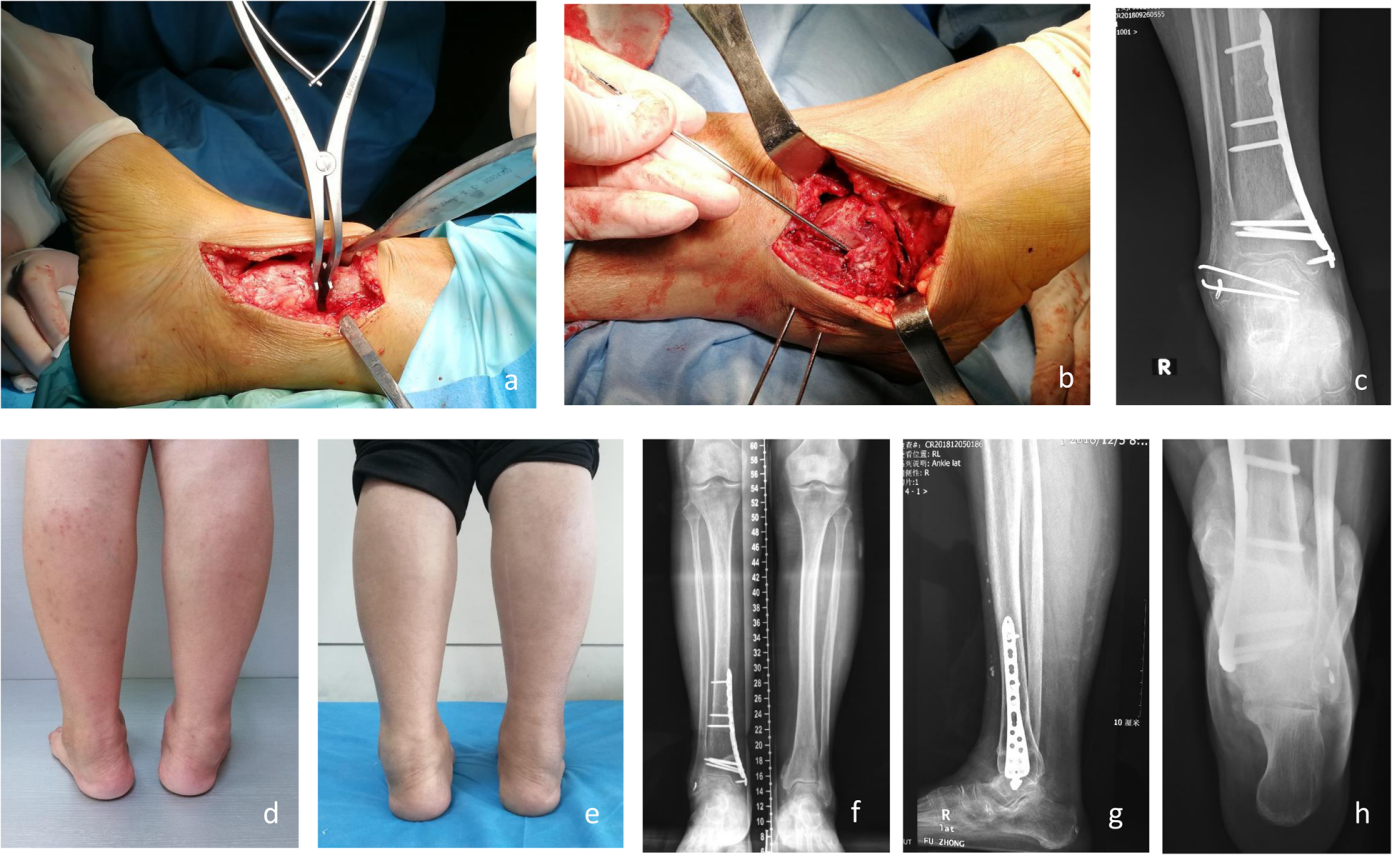
The treatment process of the same patient shown in Fig. 1. An anteromedial incision (**a**) was made for joint debridement and supramalleolar osteotomy. An anterolateral incision (**b**) was made for joint debridement, lateral ankle ligament reconstruction, and Kirschner wire fixation of the tibial talus. Anteroposterior view at 6 weeks postoperatively (**c**) showed Kirschner wire fixation of the tibial talus and good joint matching. The appearance of the right ankle before (**d**) and 20 months after surgery (**e**) showed obvious improvement of the heel alignment. At 20 months after surgery, weight-bearing anteroposterior view (**f**), lateral view (**g**), and hindfoot alignment view (**h**) showed good recovery of the MDTA, ADTA, and TTA. The TTA remained at approximately 0° and the arthritis stage improved to stage 2

**Fig. 3:**
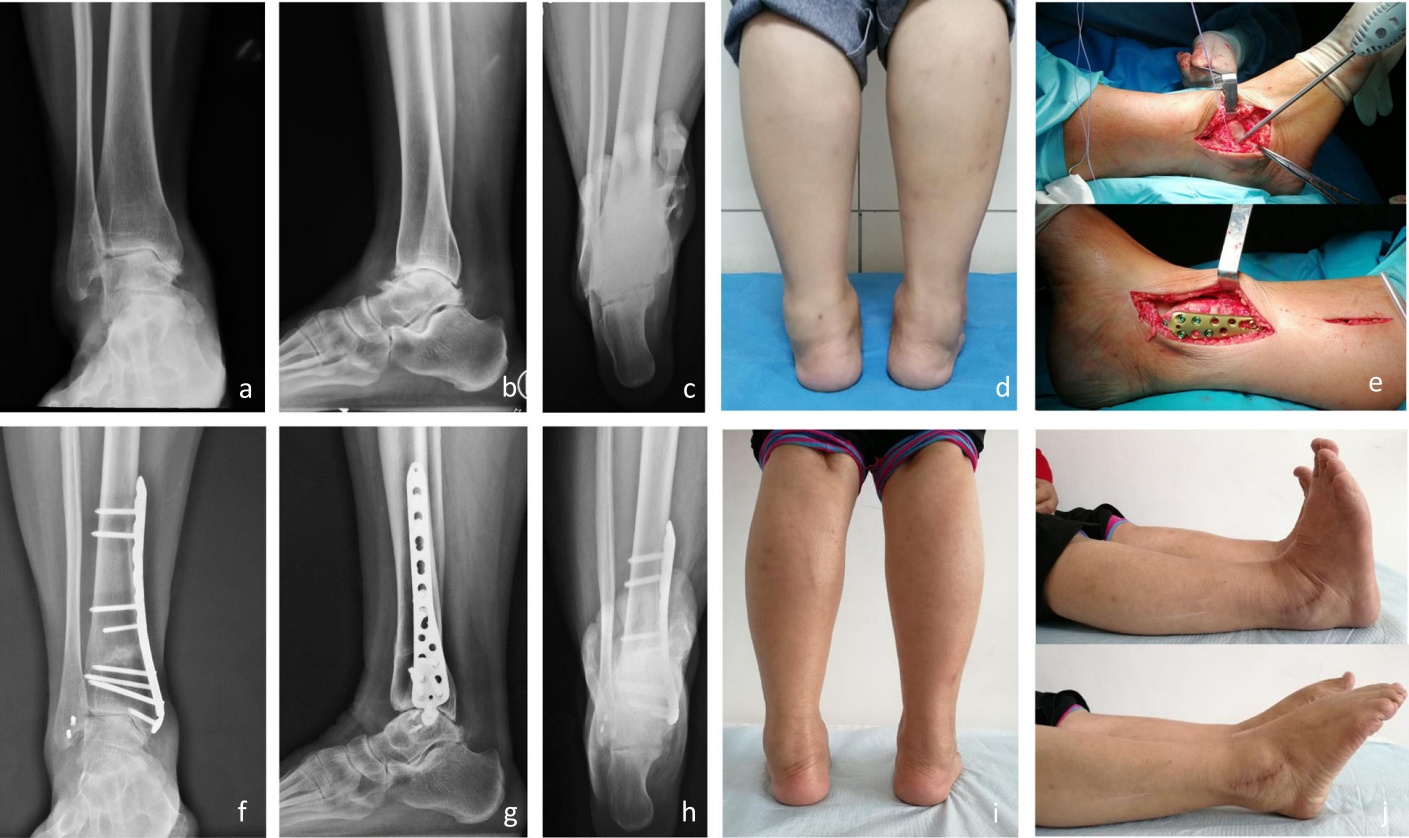
Male, 55 years old, bilateral ankle arthritis, and the right ankle was treated. Preoperative weight-bearing anteroposterior radiograph (**a**), lateral radiograph (**b**), and hindfoot alignment radiograph (**c**) of the ankle showed a stage 3a ankle arthritis. Photographs obtained on the initial examination of the patient (**d**). Intraoperative photographs showed lateral ligament reconstruction and medial supramalleolar osteotomy and plate fixation (**e**). Follow-up radiographs 2 years postoperatively (**f**–**h**) showed good recovery of the MDTA, ADTA, and TTA, and the arthritis stage improved to stage 2. The good appearance 2 years postoperatively (**i**). The extension range of motion and the flexion range of motion 2 years postoperatively (**j**)

After fixation of the distal tibia, the talus was everted to observe the medial space of the ankle, and the deltoid ligament was released if the medial gutter was narrow. Once the varus and supination deformities of the talus could be corrected passively, and the heel alignment returned to neutral, the modified Brostrom procedure [19] was performed; the lateral ankle ligaments were reinforced by imbrication with the inferior extensor retinaculum using two suture anchors. In all cases, two 2.0-mm Kirschner wires were inserted from the distal fibula into the talus body to maintain the neutral position of the talus. After the drainage tube was placed, the incision was sutured, and the elastic bandage was wrapped to reduce bleeding and prevent DVT. A typical case is shown in Figs. 2 and 3.

### Postoperative treatment

The wound drainage tube was removed approximately 48 h after surgery. The ankle was kept in a neutral position with a below-knee plaster cast for 6 weeks. Follow-up was conducted at 6 weeks, 3 months, 6 months, 1 year, and 2 years after surgery. The Kirschner wires for fixation of the lateral malleolus and talus were removed under local anesthesia 6 weeks after surgery, and partial weight-bearing movements were permitted, as tolerated. Full weight-bearing was allowed when complete bone healing was confirmed by radiography approximately 3 to 6 months after surgery.

### Clinical and radiological analysis

The AOFAS ankle-hindfoot scale was employed to assess the functional results, and a VAS score was employed to assess pain. Radiological evaluations included measurements of the MDTA, TTA, ADTA, and HMAVs. The matching of the tibia talus and the stage of the ankle joint were observed.

### Statistical analysis

All data were analyzed using SPSS version 21.0 (IBM Corp., Armonk, NY, USA). Continuous variables including the AOFAS score, VAS score, MDTA, ADTA, TTA, and HMAV were presented as the mean ± standard deviation and were analyzed using paired *t* tests. Differences were considered statistically significant at *p* ≤ 0.05.

## Results

For the group as a whole, the mean follow-up time was 32.2 months (range, 24–40 months). At the last follow-up, the AOFAS score improved significantly from 45.8 ± 2.1 (range, 35–60 points) preoperatively to 84.8 ± 1.8 (range, 66–96 points) (*p* < 0.001), and the VAS score decreased from 4.9 ± 0.4 (range, 2–8 points) to 1.1 ± 0.2 (range, 0–2 points) (*p* < 0.001).

By radiological evaluations, the MDTA, TTA, and HMAVs changed from 80.9° ± 0.4° (range, 78–83°) to 90.1° ± 0.4° (range, 86–93°), 11.7° ± 0.6° (range, 8–17°) to 1.4° ± 0.3° (range, 0–3°), and 12.6 mm ± 0.8 mm (range, 8–18 mm) to 4.2 mm ± 0.6 mm (range, 0–10 mm), respectively (each *p* < 0.001). The ADTA changed from 82.1° ± 0.3° (range, 80–84°) to 82.5° ± 0.3° (range, 80–85°), and there was no statistical significance (*p* = 0.370) (Table 1).

**Table 1:** Comparison of clinical results and radiological assessment among 17 patients before surgery and at the last follow-up

Item	Preoperative	Postoperative	Improvement	*p* value
AOFAS score	45.8 ± 2.1 (range 35–60)	84.8 ± 1.8 (range 66–96)	3.0	<0.001
VAS score	4.9 ± 0.4 (range 2–8)	1.1 ± 0.2 (range 0–2)	3.8	<0.001
MDTA (°)	80.9 ± 0.4 (range 78–83)	90.1 ± 0.4 (range 86–93)	9.2	<0.001
ADTA (°)	82.1 ± 0.3 (range 80–84)	82.5 ± 0.3 (range 80–85)	0.4	0.370
TTA (°)	11.7 ± 0.6 (range 8–17)	1.4 ± 0.3 (range 0–3)	10.3	<0.001
HMAVs (mm)	12.6 ± 0.8 (range 8–18)	4.2 ± 0.6 (range 0–10)	8.4	<0.001

Note: All values are expressed as mean ± standard deviation. Differences were considered statistically significant at *p* ≤ 0.05

*AOFAS* The American Orthopaedic Foot and Ankle Society, *VAS* visual analogue scale, *MDTA* medial distal tibial angle, *ADTA* anterior distal tibial angle, *TTA* talar tilt angle, *HMAVs* hindfoot moment arm values

All patients achieved bone healing in an average of 3.3 months (range, 3–6 months). The arthritis staging improved in 11 cases (65%); 2 cases improved from stage 2 to stage 1, 7 cases from stage 3a to stage 2, and 2 cases from stage 3b to stage 3a. No change was observed in 6 cases, and there were no aggravated cases (Table 2).

**Table 2:** Preoperative and last follow-up ankle OA staging

Last follow-up	Preoperative				
	Stage 1	Stage 2	Stage 3a	Stage 3b	Stage 4
Stage 1	0	2	0	0	0
Stage 2	0	0	7	0	0
Stage 3a	0	0	4	2	0
Stage 3b	0	0	0	2	0
Stage 4	0	0	0	0	0

Intramuscular vein thrombosis of the lower limbs occurred in 1 patient 1 week after surgery, and superficial infection occurred in 1 patient. No cases of bone nonunion, delayed union, or subsequent ankle fusion were observed during the follow-up.

## Discussion

The following parameters are significant in the evaluation of varus ankle arthritis deformities. The first parameter is MDTA. The MDTA is applied to assess the varus deformity of the distal tibia, and it varies by region. Hintermann et al. [15] reported the normal MDTA of Europeans ranged from 91 to 93°, while Monji [20] reported the mean value of Japanese was 87.7 to 89.0°. According to a biomechanical study conducted by Stufkens et al. [21], when the lateral malleolus was intact, there was no significant increase in pressure on the lateral half of the tibial talus joint when the axial load was applied after the tibial osteotomy. However, once the lateral malleolus was cut, the pressure on the lateral half of the tibial talus joint increased significantly. This suggests the necessity of considering lateral malleolus osteotomy in supramalleolar osteotomy for patients with lateral malleolus obstruction. A decreased MDTA results in varus deformity above the ankle, high pressure in the medial ankle, accelerated cartilage degeneration, and narrowed medial joint space [15]. The optimal correction with distal tibia osteotomy is a 2–4° slight overcorrection of the MDTA [20] or a correction in reference to the normal contralateral limb. With supramalleolar osteotomy, the varus deformity was effectively corrected proximal to the tibial talus joint, leading to restoration of the talar center on the axis of the tibia and dispersion of the stress on the ankle joint surface [22, 23]. In this study, the mean MDTA improved approximately 10° after the operation and was considered to be the most important reason for postoperative functional improvement.

The second parameter is TTA. The TTA is applied to assess the varus deformity of the talus on the coronal plane. An excessive TTA is often cited as one of the relative contraindications to supramalleolar osteotomy. Tanaka Y et al. [12] considered a TTA exceeding 7.3° to be a negative factor for a supramalleolar osteotomy, while Lee WC et al. [10] considered a TTA exceeding 10° to be a negative factor. A large TTA implies disability of the lateral ligament complex or an erosion of the medial malleolar subchondral bone (widening of the medial mortise space). A proper understanding of large TTAs is helpful in the formulation of targeted surgical procedures, such as arthroplasty [1, 24] for intra-articular deformity or lateral ankle ligament reconstruction [24, 25] for talar instability. It is meaningful that one of the inclusion criteria of our study was a preoperative TTA larger than 7.3°. The results showed that the TTA decreased significantly after surgery and was maintained effectively during the follow-up; this result was mainly attributed to the release of the deltoid ligament, the reconstruction of the lateral malleolar, and the fixation of the tibia and talus with Kirschner wires to maintain the reduction of the talus in the ankle mortise.

The third parameter is HMAVs [17]. The HMAV is applied to assess the varus deformity of the heel, and the average normal value is 1.6–3.2 mm [16] inside the tibial axis. The HMAV is more reliable than the tibial heel angle, which is affected by foot rotation [26]. A high HMAV in varus ankle arthritis indicates a varus deformity of the distal tibia, a decrease in the height of the medial half of the talus, or abnormal alignment of the talus and calcaneus. In rare cases, a high HMAV is also associated with varus deformity of the calcaneus body, which sometimes requires lateral sliding calcaneus osteotomy with or without a closing wedge [25]. In addition, when the talus is tilted, the calcaneus may compensate in the opposite direction, resulting in a z-shaped deformity [15]. In this situation, CT scans are recommended to identify the biplane deformity. In this study, the average HMAV changed from 12.6 mm before surgery to 4.2 mm after surgery. Although there was significant improvement, the HMAV still did not reach the normal range (1.6–3.2 mm) [17], which may be related to the coexistence of hidden subtalar joint varus or the uncorrected varus deformity of the calcaneus body.

In moderate to severe ankle osteoarthritis, approximately 60% of patients present talus varus or valgus, which may be related to talus instability [15, 27]. The stability of the talus depends on the finely matched bone structure and the healthy soft tissue around the talus, including the medial and lateral collateral ligaments, the ligaments between the talus and calcaneus, and the perimalleolar tendons. Instability of the talus can be caused by abnormal bone structure, such as erosion of the medial malleolus or dysfunction of related ligaments and tendons. In different stages, these abnormalities can exist alone or simultaneously and affect each other. A medial clear space greater than 3 mm is recommended as a predictor of widened ankle mortise on ankle stress radiographs, and a widened ankle mortise usually implies instability of the talus [1, 28].

Lateral instability of the ankle is considered to be an important cause of ankle osteoarthritis. Due to anatomical and biomechanical factors, ankle sprains are more likely to involve the lateral ligaments, which account for approximately 85% of all ankle sprains; this is consistent with the fact that varus ankle arthritis is more common than valgus ankle arthritis [1, 2, 25]. Relaxation of the anterior talofibular ligament leads to anterior displacement of the talus, which presents as ankle joint subdislocation on anterior stress views in severe cases, while a loose calcaneofibular ligament results in instability of the talus by varus stress, and the combination of the two results in a supination deformity of the talus. Lateral ankle instability can be caused by a single severe injury or multiple minor injuries, and the latter accounts for a higher proportion than the former [29]. The time course of the disease from ankle sprain to ankle joint instability to obvious osteoarthritis is approximately 20 years [30, 31], which suggests that the early treatment of lateral ankle ligament injuries should be emphasized to prevent chronic ankle joint instability and osteoarthritis. In this group, 15 patients had one or more previous obvious ankle sprains, which were believed to be related to the development of arthritis.

The correction of an excessive TTA is a difficult problem in ankle salvage surgery. Talar tilt is often accompanied by erosion of the media malleolus and widening of the medial gutter. Myerson and Zide recommend plafondplasty (intra-articular osteotomy) to narrow the mortise and reduce the TTA [32]. However, Lee believed that the procedure only corrected the medial half dome and may cause new intra-articular deformities; thus, they designed the distal tibial oblique osteotomy to correct the tilt of the entire tibial dome and to narrow the ankle mortise without damaging the joint surface and distal tibiofibular syndesmosis [14]. However, other authors considered that Lee’s technique was effective in correcting the width of ankle mortise but not effective when the TTA was too large [1].

The need for reconstruction of the lateral malleolar ligaments after distal tibial osteotomy is controversial. Lee et al. [25] and Mann et al. [24] believed that reconstruction was an essential adjunct to bony osteotomy and that the talus could be accurately stabilized in the ankle mortise. However, other authors believed that the abnormal stress within the ankle joint would be distributed after the realignment of the lower limbs, and ligament reconstruction was unnecessary [8, 9, 12]. We believe that if the treatment of ankle arthritis with a significantly increased TTA is not combined with reconstruction of the lateral ligament, the supination deformity of the talus would still exist, and the stress on the medial side of the ankle would remain too high while weight-bearing. The lateral ligament reconstruction technique (the modified Brostrom operation) used in this study is the gold standard for the treatment of CLIA; however, Lee et al. [25] showed that this technique was not effective when the TTA was too large. We considered this result to be due to the re-disability of the ligaments postoperatively. To avoid the re-relaxation of the reconstructed ligaments, we fixed the fibula and talus with two 2.0-mm Kirschner wires, immobilized the ankle joint with a short leg brace after surgery, and removed the Kirschner wires 6 weeks postoperatively. The mechanism is similar to the postoperative external fixator fixation mechanism after osteotomy introduced by Zhao et al. [33] but is more simple and minimally invasive.

Some limitations of the present study must be acknowledged. A small number of cases were included in our study, and there was no control group to confirm the positive effect of the 6-week postoperative tibia-talus fixation by Kirschner wires. In addition, the mean follow-up time was 32.2 months, but the range was large (24–40 months), which may have an impact on the efficacy results. Moreover, this study has not successfully analyzed the individual factors (high TTA, severity of arthritis preoperatively, age, etc.) that might be perhaps predictive of success or failure as a result of the sample size. Large-scale and long-term follow-up studies are needed to provide more convincing clinical data for the application of this technique.

## Conclusion

Supramalleolar osteotomy combined with lateral ligament reconstruction and talofibular immobilization can effectively improve the load of the ankle in the treatment of varus ankle arthritis. As the talar tilt angle can be significantly improved and effectively maintained, this technique could be an alternative method for the treatment of varus ankle arthritis with an excessive TTA.

## Data Availability

All data used and analyzed during this study are available from the corresponding author on reasonable request.

## Abbreviations

ADTA: Anterior distal tibial angle
AOFAS: American Orthopaedic Foot and Ankle Society
ATFL: Anterior talofibular ligament
CFL: Calcaneofibular ligament
CLIA: Chronic lateral instability of the ankle
CORA: Center of rotation of angulation
HMAVs: Hindfoot moment arm values
MDTA: Medial distal tibial angle
OA: Osteoarthritis
TTA: Talar tilt angle
VAS: Visual analogue scale
